# Epidemiological characteristics and treatment outcomes of hospitalized patients with COVID-19 in Ethiopia

**DOI:** 10.11604/pamj.supp.2020.37.1.24436

**Published:** 2020-09-11

**Authors:** Beminet Moges Gebremariam, Kalegzabher Lukas Shienka, Biruk Assefa Kebede, Mathewos Geneto Abiche

**Affiliations:** 1 Department of Public health, Wachemo University, Ethiopia,; 2 Department of Midwifery, Wachemo University, Ethiopia,; 3 Department of Biomedical science, Wachemo University, Ethiopia

**Keywords:** COVID-19, travel history, treatment outcomes, case fatality rate, ICU care quality

## Abstract

**Introduction:**

coronavirus disease (COVID-19) has been identified as the cause of an outbreak of respiratory illness which epidemiologically linked to the seafood and wet animal wholesale market in Wuhan, Hubei Province, China. Since there is paucity of research on characteristics and treatment outcomes of COVID-19, the finding of this study will helps to provide insight for the effectiveness of measures to fight against coronavirus disease in resource-limited countries.

**Methods:**

a retrospective review of released data about cases in daily bases and documents from Ethiopian public health institute website. In this article, we included and analyzed data of cases from 13 March to 13 May 2020 which were available at the time of the review.

**Results:**

a total of 263 cases were included (median age, 34 years [range, 0.9-85 years]; 76% male). COVID-19 cases among age group 15-24 years and 25-34 years were 92(35%) and 76(28.9%) respectively. More than half (55.5%) of cases had travel history abroad and African countries are the leading 64(24.3%). About 167(63.5%) cases were identified based on symptom-based surveillance and the finding confirmed an interrupted kind of epidemiological curve. Whereas, one-third (41.1%) were recovered and the overall case fatality rate was 1.9%. Four out of five patients in ICU were deceased after 2-6days spent in critical care.

**Conclusion:**

an integrated action includes the provision of health education to youths, taking measures to rise up treatment outcomes, enhancing ICU care quality. Moreover, tightening prevention and restriction measures to flattening the curve and also establishment of fast detection and advanced treatment of cases were critically requires through the patriotic efforts of frontline health workers, leaders, and stakeholders.

## Introduction

Coronavirus disease (COVID-19) has been identified as the cause of an outbreak of respiratory illness which epidemiologically linked to the seafood and wet animal wholesale market in Wuhan, Hubei Province, China beginning in December 2019 [1,2]. The incubation period of COVID-19 infection approximately 5.2 days and symptom appears after incubation period [3]. A period, 6 to 41 days with a median of 14days lapse from the onset of COVID-19 symptoms to death. Conversely, the time depends on age and immune status of patients [4]. There are few studies based on current knowledge confirmed coronaviruses are transmitted from human-to-human through respiratory fomites and respiratory viruses are most contagious when a patient is symptomatic. Then, peoples can get the infection through close contact with a person who has symptoms from the virus includes cough and sneezing [5-7]. However, there is an increasing body of evidence to suggest that human-to-human transmission may be occurring during the asymptomatic incubation period which has been estimated 2-10 days [8]. The commonest symptoms at onset of COVID-19 illness are fever, cough, and fatigue, whereas other symptoms include sputum production, headache, haemoptysis, diarrhea, dyspnea, and lymphopenia [9-12]. Globally, the number of confirmed cases as of this writing (May 13, 2020) has reached 4, 170, 424 and with total deaths of 287,399 patients [13]. In Africa based on evidence of African union member states (53) reported on 12 May, 2020 about 66,373 COVID-19 cases, 23,095 recoveries and with the deaths of 2,336 patents [14]. The first case of COVID-19 was reported in Ethiopia on 13 March, 2020 [15]. At the time of preparing this manuscript on 13 May 2020, Ethiopian Minister of Health and Ethiopian Public Health Institute reported, a total of 263 confirmed cases of COVID-19, five deaths and 108 recovered patients in Ethiopia [16]. Ethiopia is one of the countries which scaled-up public health and social measures beginning from early onset of the COVID-19 virus transmission. Despite all these efforts done by the government still in some parts of the country loosening of these prevention and control measures have been observed. There is a paucity of research on characteristics and treatment outcomes of COVID-19 cases. Therefore, this study will point out success and gaps of COVID-19 treatments in resource-limited countries and it will provide ways forward for the effectiveness of measures against coronavirus disease in Ethiopia.

## Methods

**Data source and analysis:** in the study we use extrapolated published press release of cases in daily bases as well as document from Ethiopian public health institute (EPHI) website for the public [17]. To keep the quality of the data we included data with full information based on the report and data was checked for accuracy using date and numbers. We analyzed 263 reported COVID-19 cases from 13 March to 13 May 2020 which were available at the time of the data review. We used IBM SPSS Version 25 software for the analysis.

## Results

**Demographic and geographic characteristics:** a total of 263 cases were included (median age, 34 years [range, 0.9-85 years]; 76% male). COVID-19 cases among age group 15-24 years and 25-34 years were 92(35%) and 76(28.9%) respectively. While, elderly (aged ≥60 years) accounts 18 (6.8%). Concerning sex of patents, 200(75%) were males with a male-to-female sex ratio of 3.2: 1. The majority (88.2%) of cases was Ethiopian and foreigners account, 11% on the basis of available data. The regional distribution of Coronavirus cases investigation showed that, 6 regions of 9 and two city administrations were affected by COVID-19. More than half (60.8%) of the COVID-19 cases were investigated in Addis Ababa city whereas, Oromia and Somalia regions shares the similar 19(7.2%) from the investigated cases. Therefore, coronavirus disease covered a total of 22 residential provinces in the country Table 1.

**Table 1:** demographic and geographic characteristics of hospitalized patients with COVID-19 disease in Ethiopia, from 13 March to 13 May, 2020(n=263)

Variables	Number	Percent
**Age group in yrs**		
<5	1	0.4
5-14	7	2.7
15-24	92	35.0
25-34	76	28.9
35-44	43	16.3
45-59	26	9.9
≥60	18	6.8
**Sex**		
Male	200	76
Female	63	24
**Nationality**		
Ethiopian	232	88.2
Foreigners	29	11.0
Unspecified	2	0.8
**Investigated Regions**		
Addis Ababa	160	60.8
Oromia	19	7.2
Amhara	7	2.7
SNNPR	5	1.9
Tigray	8	3.0
Somalia	19	7.2
Afar	21	8.0
Dire Dawa	7	2.7
Unspecified	17	6.5

* SNNPR: South Nation Nationality peoples Region

**Figure 1:**
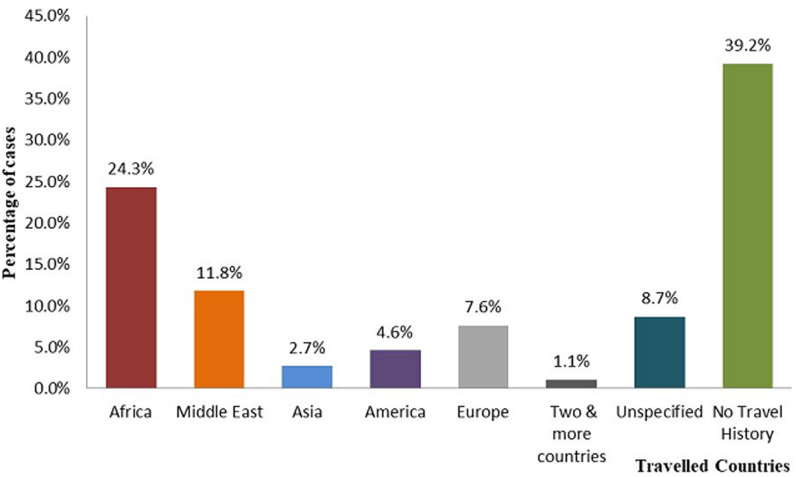
travel histories of COVID-19 Cases in Ethiopia from 13 March to 13 May, 2020

**Travel history and countries journeyed by cases:** based on the data, one hundred forty-six (55.5%) cases had travel history abroad. Although, African countries are the leading 64(24.3%) countries traveled by patients mainly East African countries accounts 61(95 %) whereas, 103 (39.2%) had no travel history Figure 1.

**Figure 2:**
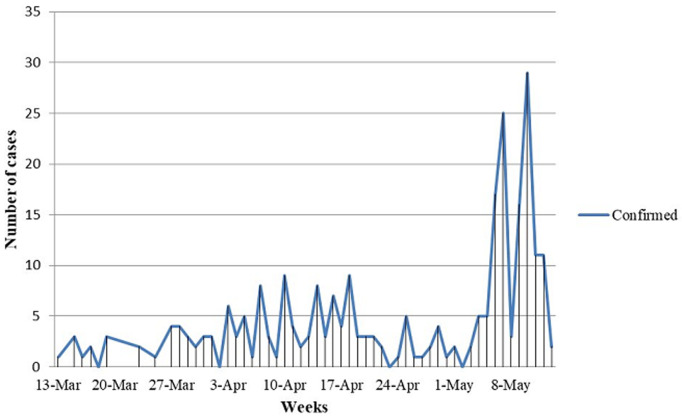
epidemiological curves of confirmed COVID-19 cases over time in Ethiopia from 13 March to 13 May, 2020

**Type of surveillance and Epidemiological curves of cases:** here from the data, about 167(63.5%) were identified based on symptom-based surveillance and 83(31.6%) of cases were detected based on contact-based surveillance. Since, the detection of index case the data confirmed cases plotted by date of diagnosis showed an interrupted propagated kind of epidemics Figure 2.

**Figure 3:**
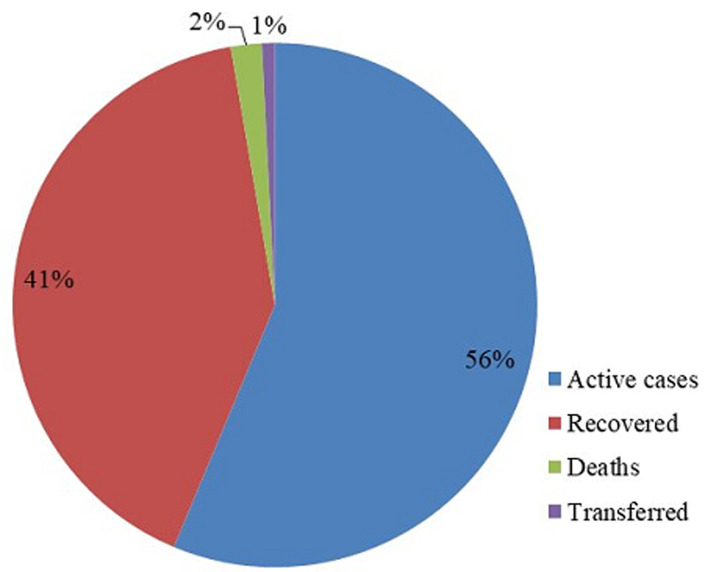
treatment outcomes of COVID-19 patients in Ethiopia from 13 March to May 13, 2020

**Treatment outcomes and case fatality rate of patients in Ethiopia:** the treatment outcomes of COVID-19 patients from 13 march to 13 may 2020 indicated that, 148(56.3%) were active cases, 108(41.1 %) recovered patients, 5(1.9%) deaths and 2(0.8%) were transferred for better treatment to their countries Figure 3. About five of ICU admitted COVID-19 Patients 80% became died, after 2-6days spent in critical care. One patient recovered from ICU. Age group 60 and above years had a case fatality rate 22.2%, followed by 3.8% on patients with age group 45-59 years Table 2.

**Table 2:** case fatality rate of COVID-19 cases in Ethiopia from 13 March to 13 May, 2020

Baseline characteristics	Confirmed cases, N (%)	Deaths, N (%)	Case fatality rate, %
Over all	263	5	1.9
**Age ,Years**			
<5	1	-	-
5-14	7	-	-
15-24	92	-	-
25-34	76	-	-
35-44	43	-	-
45-59	26	1	3.8
≥ 60	18	4	22.2
**Sex**			
Male	200	2	1.0
Female	63	3	4.8
ICU cases			
Not received	258	1	0.4
Received	5	4	80

* ICU; Intensive Care Unit

## Discussion

This analysis of early COVID-19 cases in Ethiopia provides insights about basic characteristics and treatment outcomes with in the 61days between the index case on March 13, 2020 to the end of the study May 13, 2020. The finding showed, two-thirds (64%) of cases were youths at the group 15-24 years & 25-34 years and the least was among children under five years of age 0.4%. On the bases of these findings, males were infected 3 fold than females. A systematic review and meta-analysis done by J. Yang *et al*. support the finding that, males took a larger amount than females [18]. It is customary to think women are less likely to be affected by many bacteria and viruses than are men, partly because of their more robust innate and adaptive immune responses as evidenced by research [19]. However, the finding disagreed with a study in Shenzhen China approximately equal numbers of male and female cases and also majority of coronavirus cases were adults between the ages of 30 and 69 [20]. On the other hand, majority (88.2%) of cases were Ethiopians and foreigner´s accounts, 11 percent.

Concerning the geographic distribution of novel coronavirus, it spreads rapidly from a single city to nearly 22 residential provinces of the country. Whereas, more than half of the COVID-19 cases were investigated in Addis Ababa. Based on the study, 55.5% of cases had travel history abroad. Although, African countries are the leading 24.3% countries traveled by COVID-19 patients and majority (95%) were from East African countries. However, nearly forty percent of cases had no travel history. Therefore, predominant source of infection in Ethiopia was imported coronavirus. Here from the data, nearly 65% of cases were identified based on symptom-based surveillance and contact-based surveillance detected 83(31.6%). The data of confirmed cases plotted by date of diagnosis showed that, since the first detection of index case on March 13, 2020 in Ethiopia an interrupted propagated kind of epidemics was observed with the onset of illness peaked around May 7-10, 2020. This might be due to irregularity of protective measures which includes physical distancing, quarantines, isolation and personal hygiene work to slow an epidemic by limiting the spread of the virus and flattening the curve. Other reasons might be relate with an incidence of national permission to resume transportation and social movements as the result of Christians Easter celebration from April 17-19, 2020 then after 14 days the worst hits started from first week of May. The incident was similar to study in China [21].

More than one-third (41%) of cases were recovered at the early stage of the coronavirus disease pandemic this shows substantial treatment outcomes through patriotic efforts of frontline health workers, leaders and stakeholders involved in therapeutic activities of the country. However, the result was lower than treatment outcomes by Petropoulos *et al*., which indicated 52.8% of the total confirmed cases were recovered [22]. About 80% admitted patients in Intensive Care Unit were found age 60 and above years and became deceased. But, one patient recovered after long days spent in ICU. This might be related to older patient may have severe cases and had more significant number of comorbid conditions similar to study in Wuhan, China [23]. However, on the basis of this review case fatality rate of coronavirus disease among patients aged 60 and above years was 22.2% but, the overall case fatality rate from the data was 2% which is in line with study in china showed an overall case fatality rate of 2.3% [24]. This study has several limitations. First, the researchers had little literature review since the topic is emerging. Second, the data we used was retrieved from press release with manual review from website which precluded the details so that the review missed variables such as socio economic characteristics, comorbidity and severity of cases because of inaccessibility patient records.

## Conclusion

In this study we conclude that youths, males and patients that had travel history of abroad were more infected with Coronavirus disease. More than one-third of cases were recovered and overall case fatality rate of two percent indicates substantial treatment outcomes at the early onset of the pandemic. Therefore, an integrated action includes provision of health education to youths, taking measures to rise up treatment outcomes, enhancing ICU care quality. Moreover, tightening prevention and restriction measures to flattening the curve and also establishment of fast detection and advanced treatment of cases at the border in coordination with neighboring countries and inside the country were critically requires through patriotic efforts of frontline health workers, leaders and stakeholders.

### What is known about this topic

- COVID-19 is a new pandemic viral disease;
- The incidence of COVID-19 is increasing fast and the effect may be worst in resource-limited countries including African countries;
- Epidemiological characteristics and Treatment outcomes vary across the countries.

### What this study adds

- Two-thirds (64%) of cases were youths at the group 15-34 years and males were infected 3 fold than females;
- More than one-third of cases were recovered but 80% admitted patients in ICU were deceased;
- Tightening prevention measures and establishment of fast detection and treatment of cases are seriously needed to flatten the epidemic curve.
